# Retraction Note: The apoptotic effects of soybean agglutinin were induced through three different signal pathways by down-regulating cytoskeleton proteins in IPEC-J2 cells

**DOI:** 10.1038/s41598-025-02367-3

**Published:** 2025-05-19

**Authors:** Li Pan, Tianjiao E, Chengyu Xu, Xiapu Fan, Jiajia Xia, Yan Liu, Jiawei Liu, Jinpeng Zhao, Nan Bao, Yuan Zhao, Hui Sun, Guixin Qin, Mohammed Hamdy Farouk

**Affiliations:** 1 Key Laboratory of Animal Production, Product Quality and Security, Ministry of Education, Key Laboratory of Animal Nutrition and Feed Science, Jilin Province, College of Animal Science and Technology, Jilin Agricultural University, Changchun, 130118 People’s Republic of China; 2 Animal Production Department, Faculty of Agriculture, Al-Azhar University, Nasr City, 11884 Cairo Egypt

Retraction of: *Scientific Reports* 10.1038/s41598-023-32951-4, published online 08 April 2023

The Editors have retracted this Article.

After publication, concerns were raised regarding highly similar images in Figure 4 (0.0 mg/ml SBA Keratin 8 and Keratin 18). The Authors were not able to provide the underlying raw data for this experiment upon request. In addition, the data provided for the flow cytometry analysis in Figure 1C were not consistent with the results reported in the Article.

The Editors therefore no longer have confidence in the presented data.

Li Pan disagrees with this retraction. None of the other Authors have responded to any correspondence from the Editors about this retraction.

